# Anesthetic management of carotid endarterectomy: an update from Italian guidelines

**DOI:** 10.1186/s44158-022-00052-9

**Published:** 2022-06-06

**Authors:** Sergio Bevilacqua, Giulia Ticozzelli, Massimiliano Orso, Giuseppe Alba, Laura Capoccia, Alessandro Cappelli, Carlo Cernetti, Marina Diomedi, Walter Dorigo, Gianluca Faggioli, Giovanni Giannace, David Giannandrea, Matteo Giannetta, Gianfranco Lessiani, Enrico Maria Marone, Daniela Mazzaccaro, Rino Migliacci, Giovanni Nano, Gabriele Pagliariccio, Marco Petruzzellis, Andrea Plutino, Sara Pomatto, Raffaele Pulli, Pasqualino Sirignano, Andrea Vacirca, Emanuele Visco, Shadeh Parsapour Moghadam, Gaetano Lanza, Jessica Lanza

**Affiliations:** 1 Department of Anesthesia, Azienda Ospedaliera Universitaria Careggi, Firenze, Italy; 2 Anesthesiology and Intensive Care Unit, Fondazione I.R.C.C.S. Policlinico San Matteo, Pavia, Italy; 3 Società Italiana di Chirurgia Vascolare ed Endovascolare (SICVE), Roma, Italy; 4 Department of Vascular Surgery, University of Siena, Siena, Italy; 5 Vascular and Endovascular Surgery Division, Policlinico Umberto I La Sapienza University of Rome, Rome, Italy; 6 Vascular Surgery Unit, Policlinico Le Scotte Hospital University of Siena, Siena, Italy; 7 Division of Cardiology and and Interventional Hemodynamics, Ca’ Foncello Hospital, Azienda USLL2 Marca Trevigiana, Treviso, Italy; 8 Stroke Unit, Department of Systems Medicine, Tor Vergata University Hospital, Rome, Italy; 9 Vascular Surgery Unit, University of Florence, Florence, Italy; 10 Vascular Surgery Unit, Policlinico Sant’Orsola, Alma Mater Studiorum University, Bologna, Italy; 11 Vascular Surgery Unit, Arcispedale Snata Maria Nuova, Reggio Emilia, Italy; 12 Stroke Unit, Neurology Department, USL Umbria 1, Cittá di Castello, Perugia, Italy; 13 Vascular Surgery Unit, IRCCS Policlinico San Donato Hospital University, San Donato Milanese, Italy; 14 Angiology Unit, Villa Serena Hospital, AUSL 3, Pescara, Italy; 15 Vascular Surgery Unit, Department of Policlinico Monaza, Monza, Italy; 16 Pavia University, Pavia, Italy; 17 Angiology and Internal Medicine, Valdichiana S.Margherita Hospital, Cortona, Italy; 18 Vascular Surgery Unit, Policlinico Riuniti Hospital University, Ancona, Italy; 19 Neurology and Stroke Unit, Policlinico Hospital University, Bari, Italy; 20 Stroke Unit, Marche Nord Riuniti Hospital, Fano, Italy; 21 Vascular Surgery Unit, Policlinico Careggi Hospital University, Florence, Italy; 22 Vascular and Endovascular Surgery Division, Sant’andrea Hospital , “La sapienza” University of Rome, Rome, Italy; 23 Vascular Surgery Unit, Policlinico San’Orsola-Alma Mater Studiorum University, Bologna, Italy; 24 Division of Cardiology and Interventional Hemodynamic, San Giacomo Apostolo Hospital, Azienda ULSS2 Marca Trevigiana, Castelfranco Veneto, Italy; 25 Northwick Park Hospital, London, UK; 26 Vascular Surgery Department, Multimedica Hospital-IRCCS, Castellanza, Italy; 27 Vascular Surgery Department, IRCSS Ospedale Policlinico, San Martino Genova, Italy

**Keywords:** Carotid endarterectomy, Anesthesia, Intraoperative cerebral monitoring, Heparin reversal, Blood pressure monitoring, Guidelines, Recommendations

## Abstract

**Background and aims:**

In order to systematically review the latest evidence on anesthesia, intraoperative neurologic monitoring, postoperative heparin reversal, and postoperative blood pressure management for carotid endarterectomy. The present review is based on a single chapter of the Italian Health Institute Guidelines for diagnosis and treatment of extracranial carotid stenosis and stroke prevention.

**Methods and results:**

A systematic article review focused on the previously cited topics published between January 2016 and October 2020 has been performed; we looked for both primary and secondary studies in the extensive archive of Medline/PubMed and Cochrane library databases.

We selected 14 systematic reviews and meta-analyses, 13 randomized controlled trials, 8 observational studies, and 1 narrative review. Based on this analysis, syntheses of the available evidence were shared and recommendations were indicated complying with the GRADE-SIGN version methodology.

**Conclusions:**

From this up-to-date analysis, it has emerged that any type of anesthesia and neurological monitoring method is related to a better outcome after carotid endarterectomy. In addition, insufficient evidence was found to justify reversal or no-reversal of heparin at the end of surgery. Furthermore, despite a low evidence level, a suggestion for blood pressure monitoring in the postoperative period was formulated.

**Supplementary Information:**

The online version contains supplementary material available at 10.1186/s44158-022-00052-9.

## Introduction

Carotid endarterectomy (CEA) can be performed both under general anesthesia (GA) with instrumental cerebral intraoperative monitoring and under regional anesthesia (RA) with clinical neurological monitoring [1]. In recent years, a third anesthetic modality, named cooperative patient general anesthesia (CPGA), has been added to the two aforementioned standard modalities [2]. Superiority of one anesthetic modality over another has never been demonstrated so far, in particular for what concerns postoperative events, such as stroke, myocardial infarction, and death [1].

One of the most frightening complications of CEA is perioperative stroke (Table 1) [3]. Continuous intraoperative neurological monitoring is usually indicated to limit neurological complications due to cerebral blood flow limitation caused by carotid clamping. Both **c**linical and instrumental cerebral monitoring are currently used but neither method was linked to a better outcome [1].

**Table 1:** Thirty-day CEA complications reported in the surgical arm by the NASCET group [3]

Death (1.1%)	
Disabling stroke (1.8%)	
Non-disabling stroke (3.7%)	
All stroke and death (6.5%)	
Myocardial infarction (1%)	
Wound hematoma (7.1%)	
Wound infection (2%)	
Nerve injury (8.6%)	

During CEA, heparin is administered just before carotid artery clamping. Residual heparin could cause difficult hemostasis at the end of the surgery, and postoperative bleeding complications and neck hematoma could occur. For this reason, in some centers, it is common practice to reverse heparin with protamine. However, heparin reversal could also favor thrombotic complications, and for the same reason in other centers, it is preferred not to reverse heparin. It is still unknown which of the two strategies is the best treatment [4].

Cardiovascular, cerebrovascular, or bleeding complications after CEA could be also related to postoperative arterial hypertension. Invasive or non-invasive continuous blood pressure monitoring and hypertension treatment could reduce the rate of these complications [5]. There are no indications so far on this topic but only local therapeutic protocols that allow targeted therapeutic strategies in well-defined contexts.

The Italian guidelines on diagnosis and treatment of extracranial carotid stenosis and cerebral stroke prevention have been published in October 2021 on the national guidelines section of the Italian Institute of Health site [6]. These guidelines have been drawn up through the contribution of numerous scientific societies: SICVE-Italian Society of Vascular and Endovascular Surgery, ISA-AII-Italian Stroke Association, GISE – Italian Society of Interventional Cardiology, SIAARTI- Italian Society of Anesthesia, Analgesia, Resuscitation and Intensive Care, SIAPAV-Italian Society of Angiology and Vascular Pathology, SIICP – Italian Interdisciplinary Society for Primary Care, SIRM- Italian Society of Medical and Interventional Radiology, SNAMI – National Autonomous Union of Italian Doctors, 4SSNAMI -SNAMI Scientific Society for Health, ALICE ITALIA ODV – patients association.

These guidelines also addressed these topics and can help readers to better understand the above issues.

## Methods

The present Guideline (GL) revises and updates the previous ISO-SPREAD Guideline –Cerebral Stroke: Italian guidelines for prevention and treatment- Surgical Therapy Chapter, published in 2016 [7]. The methodology applied in this update is GRADE-SIGN version [8], according to the methodological and practical indications provided by the Italian National Center for Clinical Excellence, Quality and Safety of Care [9]. The Guideline was drafted according to the AGREE reporting checklist [10].

### Members of the working group

The working group was constituted as follows: the GL Coordinator; the Scientific Technical Committee (CTS), made up of representatives of each participating Scientific Society and Patient Association; the Expert Panel, a multidisciplinary group of clinicians from the following specialties: vascular surgery, angiology, neurology, radiology, cardiology, anesthesia and intensive care, general medicine; a referent for the Methodological Group, constituted by experts in systematic literature review and evaluation of the quality of evidence; the Scientific and Technical Organizational Secretariat.

### Funding and conflict of interests

No external funding was received for the drafting of this GL. All the authors have provided their conflict of interest statements, that are available on the SICVE website (https://sicve.it/about/conflitti-di-interesse-lg-patologia-carotidea/; last access 03/03/2022). All authors have declared that they have no financial, professional or other conflicts of interest related to the topics covered in this Guideline.

### Development of PICO questions

The multidisciplinary panel of experts developed clinical questions according to the PICO model (Population, Intervention, Comparator, Outcome), according to which the recommendations were produced.

LITERATURE SEARCH, STUDY SELECTION, DATA EXTRACTION, AND QUALITY ASSESSMENT.  For each PICO or for homogeneous PICO groups, specific literature search strategies were built and launched in MEDLINE (PubMed) and in the Cochrane Library (Cochrane Database of Systematic Reviews - CDSR and Cochrane Central Register of Controlled Trials -CENTRAL), upon the period of time January 2016 – October 2020.

Two review authors independently selected the literature, based on predefined inclusion criteria related to the PICO elements and to the considered study designs. In particular, we included: secondary studies, such as systematic reviews, meta-analyses, and narrative reviews; primary studies, such as randomized controlled trials (RCT); and observational studies, both comparative (e.g., cohort studies, cross-sectional) and non-comparative (e.g. case series).

The first selection was based on title/abstract screening, while the second selection concerned the analysis of the selected articles in full text. Any discrepancy between the two authors was discussed. The methodological quality of included studies was assessed through checklists provided by the GRADE methodology -SIGN version specific to each study design of included studies (https://www.sign.ac.uk/what-we-do/methodology/checklists/, last access: 03/03/2022). Through these checklists we assessed the internal validity of the studies, evaluating their methodological quality and possible risks of bias and also the external validity, named to what extent the study results may be applied to our PICO question.

The levels of evidence attributable to the different study designs were assessed through the checklists shown in Table 2.

**Table 2:** Levels of evidence

1++	High-quality meta-analyses and systematic reviews of randomized clinical trials with very low risk of bias; single randomized clinical trials with a very low risk of bias
1+	Well-conducted meta-analyses and systematic reviews of randomized clinical trials with low risk of bias; single randomized clinical trials with low risk of bias
1-	Meta-analyses and systematic reviews of randomized clinical trials with a high risk of bias; single randomized clinical trials with a high risk of bias.
2++	High-quality systematic reviews, related to case-control or cohort studies; high-quality case-control or cohort studies with a very low risk of confounding or bias and a high probability of causality
2+	Well-conducted case-control or cohort studies with a low risk of confounding or bias and a moderate likelihood of causality
2-	Case-control or cohort studies with a high risk of confounding or bias and a significant risk of non-causality
3	Non-analytical studies, e.g., case report and case series
4	Expert opinion

After assessing the methodological quality of each article, including each PICO question, Tables of evidence were drawn, describing the main characteristics of included studies such as study design, level of evidence, population characteristics (number of patients, disease, age, sex), intervention(s), comparator(s), outcomes, measures of effect for each outcome with relative confidence intervals and *p* value, any comments regarding the methodological limits and the generalizability of the results with respect to the PICO question.

### Moving from evidence to recommendations

After evaluating the methodological quality of the included studies, for each PICO question authors filled the “Considered Judgement” which included the following nine items: (1) reliability of the studies in the body of evidence; (2) consistency of their results; (3) relevance of the studies to the target population; (4) concerns about publication bias; (5) balancing benefits and harms; (6) impact on patients; (7) feasibility in the context where the GL will be used; (8) recommendations, specifying the strength and direction, and the overall level of evidence; and (9) recommendations for research.

The panel of experts presented and discussed the Considered Judgements during plenary meetings. thereafter they proposed and shared recommendations, as an informal process of reaching consensus on wording, strength, and direction.

After balancing the desirable and undesirable effects, recommendations were formulated on four levels: strong for, weak for, weak against, and strong against; in addition, the Good Practice Points (GPP) were used to support guideline**s** users by providing indications based on experts clinical experience and, in case of scarce evidence, based on issues deemed essential to good clinical practice. Finally, when the balance of desirable and undesirable effects was uncertain, the panel made a recommendation for research.

A summary of the type of recommendations is provided in Table 3.

**Table 3:** Strength and direction of recommendations

Judgment	Recommendations
The desirable effects clearly outweigh the undesirable effects	Strong for an intervention
The desirable effects are likely to outweigh the undesirable effects	Weak for an intervention
The balance between undesirable and desirable effects is closely balanced or uncertain	Recommendation for research and for limited use within clinical trials
The undesirable are likely to outweigh the desirable effects	Weak against an intervention
The undesirable clearly outweigh the desirable effects	Strong against an intervention
Best practice recommended based on the clinical experience of the expert panel	Good Practice Point (GPP)

### Peer review

The final version of the Guidelines was sent for peer review to independent experts and representatives of patient associations, in order to receive their comments and change proposals. The reviewers were also asked to indicate any facilitating factors and obstacles to the application of the Guidelines, but also suggestions and tools for implementation.

### Guideline updating

It is planned to update the GL every three years, starting from the date of publication on the Italian National Guidelines System (SNLG).

### Methodological strengths and limitations

This guideline is based on a comprehensive literature review performed in two large electronic databases, using search strategies specific to each PICO question. The literature selection process and the quality assessment of included studies were performed by two independent authors, and this ensures the quality of the process. In formulating the recommendations, in addition to the included literature, the panel of experts considered other factors, such as the balance between advantage and disavantage, preferences of patients, and the feasibility of the recommended intervention in the Italian context.

We also acknowledge some methodological limitations. We limited our search to articles written in English, and this may have introduced a language bias. However, we are confident that the most relevant studies have been published in English. Moreover, we did not search the grey literature, such as conference proceedings and other unpublished studies.

For what concerns the quality of the studies we included in our review, although most of the systematic reviews and meta-analyses were considered of moderate/high quality, most of the RCTs and observational studies included were judged of low quality. Regarding the case series and the narrative review, they are considered the lowest level of evidence according to our methodology.

## PICO questions, evidence, and considerations

PICO questions and relative recommendations are detailed in Table 4. Search strings and authors’ evaluation of evidences are summarized in the supplementary file 1.

**Table 4:** PICO questions and recommendations

	Strength	LOE
**PICO 1**. In patient undergoing carotid endarterectomy (P), does local or locoregional anesthesia (I) compared to general anesthesia or to cooperative patient general anesthesia (C) improve the outcome (O)?		
**Recommendation 1.** In patient undergoing carotid endarterectomy, the free choice of regional anesthesia, or general anesthesia, or cooperative patient general anesthesia, is recommended, depending on the experience of the center, and patient’s preference and clinical status.	Strong for	1++
**Recommendation 2.** In patient undergoing carotid endarterectomy, it is recommended to produce further, preferably multicenter studies, to estimate the preferences and relative degrees of patient satisfaction with regards to the type of anesthesia performed: regional anesthesia, general anesthesia or cooperative patient general anesthesia.	Recommendation for research	--
**PICO 2.** In patient undergoing carotid endarterectomy (P), does clinical neurological monitoring (I), compared to instrumental neurological monitoring (C), improve the outcome (O)?		
**Recommendation 3**. In patient undergoing carotid endarterectomy, clinical or instrumental cerebral intraoperative monitoring, chosen accordingly to the type of anesthesia and the temporary shunt strategy, is recommended. Nevertheless, clinical monitoring is more sensitive.	Strong for	2+
**Recommendation 4**. In patient undergoing carotid endarterectomy, more than one instrumental monitoring method is suggested, as the association of techniques can increase the sensitivity compared to a single method.	GPP	--
**PICO 3**. In patient undergoing carotid endarterectomy (P) does heparin reversal with protamine at the end of the intervention (I), compared to no-reversal (C), improve the outcome (O)?		
**Recommendation 5**. Further studies, preferably multicenter, are recommended for patient undergoing carotid endarterectomy to assess whether intraoperative heparin reversal with protamine at the end of surgery could reduce bleeding complications without increasing the risk of thrombosis in the postoperative period.	Recommendation for research	--
**PICO 4**. In patient undergoing carotid endarterectomy (P) does postoperative blood pressure monitoring and following arterial hypertension treatment (I), compared to no monitoring (C), improve the outcome (O)?		
**Recommendation 6**. In patient undergoing carotid endarterectomy, postoperative blood pressure monitoring and the subsequent management of arterial hypertension is indicated, as it improves the outcome if compared with no blood pressure monitoring.	Weak for	2−

*LOE* level of evidence

### PICO 1

In patient undergoing carotid endarterectomy (P), does local or locoregional anesthesia (I) compared to general anesthesia or to cooperative patient general anesthesia (C) improve the outcome (O)?

#### Evidences and considerations

Literature search identified 594 publications on this topic. After screening, 14 studies were included (4 systematic reviews and meta-analyses, 4 randomized controlled trials, and 6 observational studies).

Anesthesia may play an important role for the outcome of patients undergoing CEA, given the implications between anesthesia and neurological monitoring. The need for intraoperative neurological monitoring comes from the observation that some severe perioperative strokes occur during carotid cross-clamping due to the reduction of cerebral flow below a critical level. In the NASCET study, 31.7% of severe perioperative strokes occurred intraoperatively [3]. Although only part of these events were related to cerebral blood flow impairment, signs of brain hypo-perfusion during carotid cross-clamping should be promptly recognized.

Detection of perfusion deficits is crucial to decrease the risk of adverse cerebral events. In these cases, the surgeon introduces a temporary carotid intravascular shunt, which allows restoring the blood flow, thus avoiding ischemic damage. However, shunt insertion is not without risks. Therefore, neurological monitoring should be precise, sensitive, and specific, in order to limit the use of the shunt only in selected patients in whom it is really needed.

Intraoperative clinical neurologic monitoring, which is the reference method for brain monitoring, is possible only if the patient is cooperative [11]. On the other hand, a neuroprotective effect of general anesthetic agents has been also recognized as a protective brain factor for hypo-perfusion during general anesthesia.

These drugs could improve cerebral circulation and decrease cerebral oxygen demand [12, 13]. In addition to the implications on neurological outcomes, anesthesia may also be correlated with other types of major complications that may occur post-CEA, such as perioperative cardiovascular events [14]. In this regard, a higher rate of hypotensive episodes and hemodynamic instability with subsequent strict pharmacological control has been reported for patients undergoing CEA under GA [1]. On the other hand, the higher rate of patient discomfort under RA [15] could result in a major surgical stress [16], although this could be partially relieved by sedating patients with propofol [17].

Both randomized controlled trials and meta-analysis failed to demonstrate the superiority of one method of anesthesia over the other. Therefore, indications from previous guidelines, made on the basis of a previous meta-analysis performed on 14 randomized controlled trials, still remain valid [18]. This systematic review was performed by collecting a predominant part of patients (79.1%) who were enrolled in a single randomized controlled multicenter study [1].

In this study (GALA 2008) 3.526 patients with symptomatic or asymptomatic carotid stenosis were recruited: 1.753 subjects underwent CEA under GA, while the remaining 1.773 under RA. The primary outcome of this study was 30-day occurrence of stroke, myocardial infarction or death after surgery. No significant differences were observed between the two anesthesia modalities in the combined outcome of stroke, myocardial infarction and death (4.8% in the general vs 4.5% in the local), quality of life, or hospital length of stay [1].

Since the publication of the previous Italian guidelines, two further meta-analyses have been published on this topic [19, 20]. These studies, while confirming that randomized trials were not conclusive with respect to the initial question, took into consideration also the results of non-randomized trials. This type of study had not been considered in the last Cochrane revision [18].

In this regard, the review of Harky A, et al [20], on 152.376 patients enrolled in both randomized and non-randomized trials, reported small but significant differences in favor of local anesthesia for the incidence of stroke, death, and postoperative myocardial events. Despite these differences can be attributed to bias, as it was also suggested by the authors, this should encourage further randomized trials on larger samples.

With regards to CPGA, only one randomized trial [21] on a small sample of patients has been performed comparing the CPGA associated with RA to RA alone. Another retrospective study comparing CPGA to GA [22] and one non-randomized comparative study performed on 533 consecutive patients compared with a similar historical sample treated with RA [23] have been published to date. Three other case studies are also available on CPGA [2, 24, 25]. Overall, 1.559 cases operated under CPGA have been published to date. This technique is performed with different modalities in the various centers. Nevertheless, the patient is intubated under GA in the first part of the operation whereas, during the carotid clamping, while maintaining analgesia, the depth of hypnosis is lowered in order to allow the patient’s clinical evaluation while still sedated. The excellent satisfaction of both patients [2, 21, 24] and the surgeons [2] have often been documented for this technique. CPGA could combine the advantages of the two standard anesthetic techniques, minimizing their disadvantages [2]. In fact, as with RA, CPGA allows the clinical neurological monitoring of the patient during carotid clamping, but like GA, it allows the absolute control of the airways and ventilation. Thus, conversion to GA, if needed, is simple, quick, and relatively free from risk. In addition, the possibility to monitor anesthesia during carotid cross-clamping could reduce on one hand psychological and surgical stress, and on the other hand may ensure a favorable hemodynamic control if compared to RA [2]. The incidence of both neurological and cardiac complications reported for CPGA is in line with those documented in larger studies performed on GA and RA. Nonetheless, any definitive conclusions about the outcomes can be reached given the small number of patients examined and the observational nature of the aforementioned studies.

In conclusion, since there is any evidence that the choice of anesthesia technique is correlated with a better outcome, each surgical team can choose the type of anesthesia according to the preferences of the institution and the patient.

Moreover, in order to explore patients’ point of view regarding the choice of anesthesia for CEA, patient satisfaction and preference for the various anesthesia techniques were also investigated. In particular, the study of Mracek J et al [15]. on 159 patients operated under GA and 30 patients operated under RA was examined. This study concluded that although the level of patient satisfaction is high for both standard anesthesia techniques, satisfaction about anesthesia [AG: 148 pts (93.1%); ALR 30 (65.2%) *p*< 0.0001] and preference for the same type of anesthesia in a future operation [AG: 154 pts (96.9%); ALR: 28 pts (60.9%) *p*< 0.0001] were significantly higher for GA. Bevilacqua et al. [2], by administering to their patients a questionnaire on the day after surgery, reported higher patient satisfaction with CPGA [very satisfied: 112 pts. (61.87%); satisfied: 67 pts (37.01%); dissatisfied: 2 pts (1.1%); very dissatisfied: 0].

In conclusion, the small number of patients in the examined studies and their retrospective nature prevent us to reach definite results on this subject to date. Therefore, the panel encouraged further multicenter studies with the active involvement of patient associations, to appropriately investigate this topic.

#### Recommendation 1

In patient undergoing carotid endarterectomy, free choice of regional anesthesia, general anesthesia, or cooperative patient general anesthesia is recommended, depending on the experience of the center, patient’s preference, and clinical status (strong for, level of evidence 1++).

#### Recommendation 2

In patient undergoing carotid endarterectomy, it is recommended to produce further, preferably multicenter studies, with the aim to estimate the preferences and relative degrees of patient satisfaction with regards to the type of anesthesia performed: regional anesthesia, general anesthesia or cooperative patient general anesthesia (recommendation for research).

### PICO 2

In patient undergoing carotid endarterectomy (P), does clinical neurological monitoring (I), compared to instrumental neurological monitoring (C), improve the outcome (O)?

#### Evidences and considerations

Literature search identified 271 publications on this topic. After the screening process**,** 8 studies were included (6 systematic reviews and meta-analyses, and 2 observational studies).

Awake patient’s clinical neurological monitoring is universally considered as more reliable and sensible in detecting cerebral ischemia than any instrumental method, given the high sensitivity of the nervous system even to short periods of ischemia or hypoxia. It is universally agreed that clinical monitoring has sensitivity and specificity close to 100% in rapidly identifying the onset of a new brain perfusion defect. Therefore, this is currently considered as the reference method for comparison with any other instrumental neurological method [26].

However, awake patient’s clinical neurological evaluation is feasible only if the patient is collaborating under regional anesthesia (RA) when the patient is fully awake. It was reported that this is also feasible if regional anesthesia is associated to a variable degree of sedation and analgesia, as under cooperative patient general anesthesia (CPGA) [2].

Conversely, awake patient’s clinical monitoring is not possible under general anesthesia (GA) or whenever RA or CPGA have to be converted to GA. For this reason, anesthesia and neurological monitoring for CEA are strongly interlinked.

The neurological instrumental monitoring methods that can be used as an alternative or in association with clinical monitoring are essentially represented by:

- Electroencephalography (EEG) [27]
- Somatosensory evoked potentials (SSEPs) [28] and/or motors evoked potentials (MEPs) [20, 29]
- Stump pressure (SP) [30]
- Transcranial Doppler (TCD) [31]
- Cerebral near-infrared spectroscopy (cerebral NIRS) [32];

Electroencephalography (EEG) is a complex method, difficult to interpret, and often requires specific technical support. In addition, a meta-analysis conducted by Guay J, et al. [33] on 742 patients demonstrated 70% sensitivity and 96% specificity in EEG detecting episodes of cerebral ischemia during CEA. Another prospective study conducted on collaborating patients operated on CEA confirmed the occurrence of a high incidence of false negatives with EEG (39%), although low incidence of false positives was also reported (1%). However, it should be considered that these findings are not directly transferable to patients under general anesthesia, as in this condition many factors can affect the EEG trace and its interpretation [26].

In addition, EEG processes only the cortical electrical activity while it doesn’t detect changes in the deeper brain structures. A more recent meta-analysis collecting data from 8.765 patients in a single randomized trial and several other retrospective and prospective cohort studies, found an even lower sensitivity for EEG (52%), which in the authors' opinion would prevent using this monitoring mode as the only control method during carotid clamping [34]. Similar conclusions come also from another even more recent meta-analysis that reported 46% sensitivity and 86% specificity for EEG [35].

Somatosensory evoked potentials (SSEPs) record changes in electroencephalographic activity in response to sensory stimuli. Therefore, unlike EEG, this method explores also deeper brain structures. Nevertheless, a meta-analysis conducted by Nwachuku EL et al. [31], including only retrospective and prospective cohort studies, showed that SSEP have 58% sensitivity and 91% specificity, in order to selectively identify the need to insert the shunt during CEA reporting a 0.6% false-negative rate. However, this method is incomplete, as it investigates only the nerve pathways involved in somatosensory responses. Moreover, taking advantage of electroencephalography, many factors can affect the interpretation of data in patients who are operated under GA.

Similarly, even motor evoked potentials (MEPs) explore only partially brain activity. This method selectively investigates the nerve pathways involved in motor responses. For this reason, in some studies, they were considered in addition to SSEP as a useful tool to increase the sensitivity of this last method. In fact, in a multicenter retrospective observational study of 600 patients undergoing CEA with both methods, MEP detected 1.5% of patients at risk of cerebral hypoperfusion, while SSEP failed to detect them [29].

The measurement of arterial pressure distally to the clamp, the so-called stump pressure (SP) is another widely used method. It has been used alone or in combination with other methods to decide whether to selectively insert the endovascular shunt [26]. This pressure is directly proportional to the magnitude of retrograde flow guaranteed by the Willis circle when the carotid artery is clamped. However, the critical threshold of SP is still unknown. The value, indicated in various studies, varies between 25 and 70 mmHg. Regardless of this obvious limitation, the meta-analysis of Guay J et al [33]. reported a 75% sensitivity and 88% specificity for this method. Even more recently, another meta-analysis concluded that SP alone, whatever the pressure range is chosen, is not a reliable parameter to reduce the incidence of TIA, stroke, and death during CEA [36]. In recent years, only one small, randomized trial was published comparing routine with selective shunting if SP was less than 40 mmHg. The author concluded that there were no significant differences between the two strategies. Therefore, this study does not add useful data in response to our clinical question [37].

Transcranial Doppler (TCD) measures both the Doppler flow and embolic signals in the middle cerebral artery. Flow reduction is proportional to the decrease of cerebral perfusion.

For this method, similarly to SP, different thresholds of flow reduction were proposed to suggest the selective shunt insertion. The meta-analysis of Guay J et al. [33], in spite of different cut-off values considered in the included studies, reported 81% sensitivity and 92% specificity of TCD in detecting critical cerebral hypo-perfusion. This data resulted from studies in which TCD was used together with clinical monitoring in the awake patient undergoing regional anesthesia. Noteworthy, the frequent inadequate acoustic window could limit TCD imaging to 80-90% of cases. An undeniable advantage of this method is its unique ability to simultaneously detect the occurrence of cerebral embolism together with cerebral hypoperfusion [38]. A recent review, reported 56% sensitivity and 72.7% specificity of TCD both for detecting critical blood flow velocity variations in median cerebral artery and for identifying micro embolic signals [39].

Cerebral near-infrared spectroscopy (cerebral NIRS) can also detect cerebral hypo-perfusion in clamped carotid artery side showing a decrease in saturation from the values observed before clamping and from the contralateral side. Conflicting data have been reported for this method and the critical threshold of saturation decrease is still unclear. Thus, the reported sensitivity and specificity of this method both increase and decrease when a lower threshold is selected. Overall, Guay J et al. reported a 74% sensitivity and 82% specificity of NIRS, collecting data from retrospective studies on awake patients [33].

In conclusion, from the available literature every method of instrumental neurological monitoring appear to demonstrate lower sensitivity with respect to clinical evaluation of the awake patient. Nevertheless, there are not enough robust data to indicate the preference of a specific instrumental monitoring method for CEA. Therefore, the panel, according to a previous Cochrane review concluded that there is no reliable evidence to date, to support the selective use of the shunt in patients treated under general anesthesia, and there is still little evidence to support the use of one form of instrumental monitoring compared to another. Further studies are needed to come to definite conclusions [40].

Nonetheless, the panel still wanted to suggest intraoperative neurological monitoring, in any form, believing that it is essential for the safe execution of CEA. Moreover, it was highlighted that clinical monitoring is more sensitive than any other instrumental monitoring method.

Furthermore, some studies suggested that the association of multiple diagnostic modalities may improve the sensitivity of the single strategy. In addition to the aforementioned study of Malcharek MJ, which described the association of SSEP and MEP [29], another retrospective observational study on 1.165 patients, suggested that the association of SSEP and EEG could increase the sensitivity of the two methods when used alone [41]. However, the observational nature of these studies and the small samples, prevented us to reach definite conclusions. The limited sensitivity offered by each of the considered instrumental techniques, would favor the choice of a multimodal approach especially when a selective shunt strategy is preferred.

#### Recommendation 3

In patient undergoing carotid endarterectomy, clinical or instrumental cerebral intraoperative monitoring, chosen accordingly to the type of anesthesia and the temporary shunt strategy, is recommended. Nevertheless, clinical monitoring is more sensitive (strong for, level of evidence 2+).

#### Recommendation 4

In patient undergoing carotid endarterectomy, more than one instrumental monitoring method is suggested, as the techniques association can increase the sensitivity compared to a single method (GPP).

### PICO 3

In patient undergoing carotid endarterectomy (P), does heparin reversal with protamine at the end of the intervention (I), compared to no-reversal (C), improve the outcome (O)?

#### Evidences and recommendations

Literature search identified 37 publications on this topic. After the screening process 3 studies were included (2 systematic reviews and meta-analyses and 1 randomized controlled trial).

Only one small, randomized study has been performed on this topic (64 patients). It concluded that heparin reversal at the end of surgery reduced postoperative bleeding. In the same study, two lethal cases of cerebral thrombosis were detected in the group subjected to reversal, while two cases of hematoma occurred in the group in which reversal had not been carried out and required a surgical re-exploration [4].

A recent meta-analysis explored the pros (reduction of bleeding complications) and cons (increase in thrombotic complications) of the two different strategies. This review analyzed mostly retrospective studies and included 10,621 patients. No significant difference in stroke, cardiovascular events and mortality between the two strategies was reported. In contrast, heparin reversal with protamine showed a 43% reduction in the risk of bleeding [42].

Another meta-analysis by Kasisis, et al, including 7 retrospective studies on about 10,000 patients was performed to evaluate the safety and efficacy of heparin reverse with protamine at the end of CEA [43]. Heparin reversal with protamine was performed in 3.817 pts, while in the remaining 6.070 pts heparin was not reversed. This author demonstrated a lower rate of neck hematoma in the protamine group (1.1% vs 3.6% in the non-reverse group). In contrast, there was no significant difference between the two groups in the incidence of stroke (1.3% in the protamine group vs. 1.8% in the non-protamine group).

Although both these reviews showed a lower bleeding complications rate in patients undergoing CEA when heparin was reversed with protamine, without increasing the risk of thrombotic complications, the retrospective nature of the collected data prevented us to reach a definitive conclusion. The aforementioned meta-analysis mostly reported data from observational studies, which in most cases do not take into account the dosage of both heparin and protamine, and the concomitant antiplatelet therapy.

In conclusion, we could not find sufficiently robust data to make a recommendation in favor of one of the two strategies.

#### Recommendation 5

Further studies, preferably multicenter, are recommended for patient undergoing carotid endarterectomy to assess whether intraoperative heparin reversal with protamine at the end of surgery could reduce bleeding complications without increasing the risk of thrombosis in the postoperative period (Recommendation for research).

### PICO 4

In patient undergoing carotid endarterectomy (P), does postoperative blood pressure monitoring and following arterial hypertension treatment (I), compared to no monitoring (C), improve the outcome (O)?

#### Evidences and considerations

Literature review identified 32 publications on this topic. After the screening process, 10 studies were included (2 systematic reviews and meta-analyses, 7 observational studies, and 1 narrative review).

To address the question, a literature review was carried out until October 2020. The research was extended for a decade, since the previous 2016 Italian guidelines did not consider this subject. The way in which post-CEA hypertension occurs is still not well understood. Several hypotheses were proposed over the last few decades. The increased secretion of norepinephrine inside the brain or a baroreceptor dysfunction linked to the manipulation of the carotid sinus and/or plaque removal have been proposed. An analysis of several risk factors in 221 patients showed a higher rate of postoperative hypertension in patients who had myocardial infarction, coronary angioplasty and statin therapy [44].

On the other hand different surgical techniques used for CEA were associated to this occurrence. In a recent meta-analysis, postoperative hypertension was mainly found after the eversion technique [45]. The reason for this finding could be the loss of baroreceptor reflex, linked to the transection of the sensory tissue, and the subsequent deregulation of negative feedback operated by the carotid baroreceptor [46]. Conflicting results were obtained by other authors who did not demonstrate the association between the surgical technique and the rate of post-CEA hypertension in another group of 560 patients. These authors identified preoperative arterial hypertension as a risk factor [47].

A similar result was reported by Newman in a study of 106 patients undergoing CEA under general anesthesia. He found that postoperative hypertension was associated with poor preoperative blood pressure control and altered baroreceptor sensitivity [48]. In the same group of patients, analysis of intraoperative variables, such as hypotension at the induction of general anesthesia, use of vasoconstrictors, postoperative pain scale, velocities in the median cerebral artery recorded with transcranial doppler were not a predictive factor of postoperative hypertension [49].

Undoubtedly, pre-operative hypertension is the most predictive risk factor for the onset of postoperative hypertension, with a variable rate, depending on the considered pressure threshold value (systolic pressure value between 150 and 180 mmHg) [50].

Post-CEA hypertension was also found to be a risk factor for the development of Cerebral Hyperperfusion Syndrome (CHS) when it was associated with some preoperative risk factors such as diabetes, advanced age, recent carotid revascularization (<3 months), and severe contralateral stenosis. This is a rare complication reported post-CEA in 1% of cases [50, 51]. and a potentially preventable clinical scenario, defined as focal brain damage occurring post carotid revascularization. Criteria that define this syndrome are:

1. Onset within 30 days from surgical procedure,
2. Headache: convulsions, hemiparesis, and coma associated with radiological signs including cerebral edema or cerebral hemorrhage;
3. Evidence of hyperperfusion at radiological imaging or systolic blood pressure above 180 mmHg;
4. No evidence of new cerebral ischemia, postoperative carotid thrombosis, and possible pharmacological/metabolic causes.

The pathophysiology of CHS involves both impaired regulation of the cerebral vascular system and postoperative hypertension [50].

No CHS has been reported with systolic blood pressure below 135 mmHg, while it has been shown that the incidence of this syndrome is higher when systolic blood pressure is higher than 150 mmHg [50].

Therefore, based on these data, postoperative strategies aimed to treat arterial hypertension were proposed, with the aim of defining pressure thresholds, timing, and therapy of post-CEA hypertension both in asymptomatic patients (high blood pressure without related symptoms) and symptomatic (high blood pressure associated with clinical symptoms).

Naylor showed that the application of postoperative hypertension monitoring and treatment protocols was effective in preventing the incidence of stroke/death associated with carotid surgery, analyzing 21 years of audits aimed at finding preventive strategies for post-CEA perioperative stroke [5, 52]. This was a retrospective study that analyzed the outcomes of 2.300 patients undergoing CEA over a long time frame during which there was a significant reduction in postoperative neurological complications. Authors considered this result to be related to an improvement in surgical patient management protocols in the perioperative period that included blood pressure control. However, given the observational nature of this report, several biases cannot be excluded. Decrease in the rate of perioperative stroke could be the result of a multimodal strategy (early initiation of antiplatelet therapy and statins, in addition to the aforementioned blood pressure control) and of the improvement in the surgical technique over the long-time interval too.

In conclusion, although the robustness of these studies is not high enough, specifically for CEA, experts opinion is to suggest postoperative monitoring of blood pressure. This suggestion arouses from other many evidences that link postoperative hypertension to major complications in other surgical settings [53, 54], in particular for CEA with the above-cited CHS [50].

#### Recommendation 6

In patient undergoing carotid endarterectomy, postoperative blood pressure monitoring and the subsequent management of arterial hypertension is indicated, as it improves the outcome if compared with no blood pressure monitoring (weak for, level of evidence 2-).

## Supplementary Information

**Additional file 1.**

## Data Availability

Not applicable
