# Supplementary material for: Anesthetic management of carotid endarterectomy: an update from Italian guidelines
Source: J Anesth Analg Crit Care. 2022 Jun 6;2:24. doi: 10.1186/s44158-022-00052-9 (PMC10245611; doi:10.1186/s44158-022-00052-9)
Supplement: Supplementary file 1 — Additional file 1. [file 44158_2022_52_MOESM1_ESM.docx]

**SUPPLEMETARY DATA**

**SEARCH STRINGS:**

**PICO 1**

| PUBMED SEARCH, 3/10/2020. Comparison between LA and GA | | |
| --- | --- | --- |
| N. | Search strings | Results |
| #1 | "local anesthesia"[Tiab] OR local[Tiab] OR anesthesia[Tiab] OR anaesthesia[Tiab] | 868.049 |
| #2 | "regional anesthesia"[Tiab] OR regional[Tiab] OR anesthesia[Tiab] OR anaesthesia[Tiab] | 471.024 |
| #3 | "general anesthesia"[Tiab] OR general[Tiab] OR anesthesia[Tiab] OR anaesthesia[Tiab] | 1.131.831 |
| #4 | "carotid endarterectomy"[Tiab] OR carotid[Tiab] OR endarterectomy[Tiab] | 123.920 |
| #5 | #1 OR #2 | 1.080.093 |
| #6 | #5 AND #3 | 272.659 |
| #7 | #6 AND #4 | 3.256 |
| #8 | #6 AND #4 Filters: English, from 2016 - 2020 | 474 |

| COCHRANE SEARCH, 7/10/2020. Comparison between local and regional anesthesia | | |
| --- | --- | --- |
| N. | Search strings | Results |
| #1 | (carotid endarterectomy):ti,ab,kw (Word variations have been searched) | 1.871 |
| #2 | (anesthesia):ti,ab,kw (Word variations have been searched) | 76.820 |
| #3 | #1 AND #2 (Word variations have been searched) | 295 |
| #4 | #1 AND #2 with Cochrane Library publication date Between Jan 2016 and Dec 2020 | 66 |

| PUBMED SEARCH, 10/10/2020. Comparison between GA or RA and CPGA | | |
| --- | --- | --- |
| N. | Search strings | Results |
| #1 | "local anesthesia"[Tiab] OR local[Tiab] OR anesthesia[Tiab] OR anaesthesia[Tiab] | 869.082 |
| #2 | "regional anesthesia"[Tiab] OR regional[Tiab] OR anesthesia[Tiab] OR anaesthesia[Tiab] | 471.524 |
| #3 | "general anesthesia"[Tiab] OR general[Tiab] OR anesthesia[Tiab] OR anaesthesia[Tiab] | 1.131.831 |
| #4 | "carotid endarterectomy"[Tiab] OR carotid[Tiab] OR endarterectomy[Tiab] | 123.920 |
| #5 | "preserved consciousness"[Tiab] OR "wake-up"[Tiab] OR cooperat*[Tiab] OR combin*[Ti] OR asleep[Tiab] OR "awake test"[Tiab] | 419.870 |
| #6 | #1 OR #2 | 1.081.389 |
| #7 | #6 AND #3 | 272.903 |
| #8 | #7 AND #4 | 3.259 |
| #9 | #8 AND #5 | 93 |
| #10 | #8 AND #5 Filters: English, from 2016 - 2020 | 22 |

| COCHRANE SEARCH, 10/10/2020. Comparison between GA or RA and CPGA | | |
| --- | --- | --- |
| N. | Search strings | Results |
| #1 | (carotid endarterectomy):ti,ab,kw (Word variations have been searched) | 1.871 |
| #2 | (anesthesia):ti,ab,kw (Word variations have been searched) | 76.820 |
| #3 | (preserved consciousness):ti,ab,kw OR (wake-up):ti,ab,kw OR (cooperat*):ti,ab,kw OR (combin*):ti,ab,kw OR (asleep):ti,ab,kw OR (awake test):ti,ab,kw (Word variations have been searched) | 267.094 |
| #4 | #1 AND #2 (Word variations have been searched) | 247 |
| #5 | #3 AND #4 | 55 |
| #6 | #3 AND #4 with Cochrane Library publication date Between Jan 2016 and Dec 2020 | 21 |

LA= locoregional anesthesia; GA=General anesthesia; CPGA=Cooperative patient general anesthesia

**PICO 2**

| PUBMED SEARCH, 7/10/2020. Comparison among neurological monitoring techniques | | | |
| --- | --- | --- | --- |
| N. | Search strings | Results |  |
| #1 | "carotid endarterectomy"[Tiab] | 10.565 |  |
| #2 | monitor*[Tiab] OR EEG[Tiab] OR Electroencephalography[Tiab] OR TCD[Tiab] OR SSEP[Tiab] OR NIRS[Tiab] OR stump[Tiab] | 907.816 |  |
| #3 | #1 AND #2 | 1.332 |  |
| #4 | #1 AND #2 Filters: English, from 2016 - 2020 | 197 |  |

| COCHRANE SEARCH, 7/10/2020. Comparison among neurological monitoring techniques | | | |
| --- | --- | --- | --- |
| N. | Search strings | Results |  |
| #1 | (carotid endarterectomy):ti,ab,kw (Word variations have been searched) | 1.871 |  |
| #2 | (EEG):ti,ab,kw OR (SSEP):ti,ab,kw OR (TCD):ti,ab,kw OR (NIRS):ti,ab,kw OR (doppler):ti,ab,kw OR (Electroencephalogram):ti,ab,kw OR (monitor):ti,ab,kw OR (stump):ti,ab,kw (Word variations have been searched | 101.380 |  |
| #3 | #1 AND #2 (Word variations have been searched) | 166 |  |
| #4 | #1 AND #2 with Cochrane Library publication date Between Jan 2016 and Dec 2020 | 72 |  |

**PICO 3**

| PUBMED SEARCH, 7/10/2020. Comparison between heparin neutralizzation and non-neutralizzation | | | |
| --- | --- | --- | --- |
| N. | Search strings | Results |  |
| #1 | "carotid endarterectomy"[Tiab] | 10.568 |  |
| #2 | (reverse*[Tiab] AND (anticoagul*[Tiab] OR heparin)) OR protamine[Tiab] | 10.701 |  |
| #3 | #1 AND #2 | 61 |  |
| #4 | #1 AND #2 Filters: English, from 2013 - 2020 | 26 |  |

| COCHRANE SEARCH, 7/10/2020. Comparison between heparin neutralizzation and non-neutralizzation | | | |
| --- | --- | --- | --- |
| N. | Search strings | Results |  |
| #1 | (carotid endarterectomy):ti,ab,kw (Word variations have been searched) | 1.871 |  |
| #2 | (anticoagul*):ti,ab,kw OR (heparin):ti,ab,kw)) (Word variations have been searched) | 21.555 |  |
| #3 | (revers*):ti,ab,kw (Word variations have been searched) | 25.996 |  |
| #4 | (Protamine) :ti,ab,kw (Word variations have been searched) | 1.032 |  |
| #5 | #2 AND #3 | 645 |  |
| #6 | #4 OR #5 | 1.556 |  |
| #7 | #1 AND #6 | 11 |  |

**PICO 4**

| PUBMED SEARCH, 2/11/2020. Comparison between postoperative blood pressure monitoring and non-monitoring | | | |
| --- | --- | --- | --- |
| N. | Search strings | Results |  |
| #1 | carotid endarterectomy AND postoperative hypertension | 464 |  |
| #2 | carotid endarterectomy AND postoperative hypertension  Filters: from 2010-2020 | 139 |  |
| #3 | carotid endarterectomy AND postoperative hypertension  Filters: Clinical Trial, Meta-Analysis, Randomized Controlled Trial, Systematic Review | 37 |  |
| #4 | #2 AND 3 | 13 |  |

| COCHRANE SEARCH, 2/11/2020. Comparison between postoperative blood pressure monitoring and non-monitoring. | | | |
| --- | --- | --- | --- |
| N. | Search strings | Results |  |
| #1 | carotid endarterectomy AND postoperative hypertension | 56 |  |
| #2 | carotid endarterectomy AND postoperative hypertension  Filters from 2010-2020 | 19 |  |
| #3 | carotid endarterectomy AND postoperative hypertension  Filters: Reviews, Protocols, Trials | 19 |  |
| #4 | #2 AND # 3 | 19 |  |

**AUTHORS’ EVALUATION OF EVIDENCES**

**PICO 1**

| Evaluation of evidences. Comparisons among anesthesia techniques | |
| --- | --- |
| The following papers have been selected. In detail:  n. 4 Systematic review and meta-analysis  n. 4 Randomized controlled trials  n. 6 Observational studies or case series | Level of evidence |
| **Sistematic review and meta-analysis (n. 4)** | |
| Vaniyapong T, Chongruksut W, Rerkasem K (2013) Local versus general anaesthesia for carotid endarterectomy. Cochrane Database Syst Rev 19:CD000126. | 1++ |
| Rerkasem K, Rothwell PM (2008) Local versus general anaesthesia for carotid endarterectomy. Cochrane Database Syst Rev (4):CD000126. | 1- |
| Harky A, Chan JSK, Kot TKM, Sanli D, Rahimli R, Belamaric Z, Ng M, Kwan IYY, Bithas C, Makar R, Chandrasekar R, Dimitri S (2020) General anesthesia Versus local anesthesia in carotid endarterectomy: a systematic review and meta-analysis. J Cardiothorac Vasc Anesth 34:219-234. | 1+ |
| Hajibandeh S, Hajibandeh S, Antoniou SA, Torella F, Antoniou GA (2018) Meta-analysis and trial sequential analysis of local vs. general anaesthesia for carotid endarterectomy. Anaesthesia 73:1280-1289. | 1+ |
| **Randomized controlled trials (n.4)** | |
| GALA Trial Collaborative Group, Lewis SC, Warlow CP, Bodenham AR, Colam B, Rothwell PM, Torgerson D, Dellagrammaticas D, Horrocks M, Liapis C, Banning AP, Gough M, Gough MJ (2008) General anaesthesia versus local anaesthesia for carotid surgery (GALA): a multicentre, randomised controlled trial. Lancet 372:2132-2142. | 1++ |
| Luchetti M, Canella M, Zoppi M, Massei R. Comparison of regional anesthesia versus combined regional and general anesthesia for elective carotid endarterectomy: a small exploratory study. Reg Anesth Pain Med. 2008;334:340-345. | 1- |
| Takolander R, Bergqvist D, Hulthén UL, Johansson A, Katzman PL (1990) Carotid artery surgery. Local versus general anaesthesia as related to sympathetic activity and cardiovascular effects. Eur J Vasc Surg 4:265-270. | 1- |
| Szabó P, Mayer M, Horváth-Szalai Z, Tóth K, Márton S, Menyhei G, Sínay L, Molnár T (2020) Awake sedation with propofol attenuates intraoperative stress of carotid endarterectomy in regional anesthesia. Ann Vasc Surg 63:311-318. | 1- |
| **Observational studies or case series (n.6)** | |
| Marinò V, Aloj F, Vargas M, Spinelli G, Pompeo F, Chiacchiari L, Servillo G, Franco E (2018) Intraoperative neurological monitoring with evoked potentials during carotid endarterectomy versus cooperative patients under general anesthesia technique: a retrospective study. J Neurosurg Anesthesiol 303:258-264. | 2- |
| Bevilacqua S, Romagnoli S, Ciappi F, Lazzeri C, Gelsomino S, Pratesi C, Gensini GF (2009) Anesthesia for carotid endarterectomy: the third option. Patient cooperation during general anesthesia. Anesth Analg 1086:1929-1936. | 3 |
| Mracek J, Kletecka J, Holeckova I, Dostal J, Mrackova J, Mork J, Priban V (2019) Patient satisfaction with general versus local anesthesia during carotid endarterectomy. J Neurol Surg A Cent Eur Neurosurg 80:341-344. | 2- |
| Coppi G, Moratto R, Ragazzi G, Nicolosi E, Silingardi R, Benassi Franciosi G, Rambaldi M, Navi A, Ciardullo AV (2005) Effectiveness and safety of carotid endarterectomy under remifentanil. J Cardiovasc Surg 464:431-436. | 2- |
| Baldinelli F, Pedrazzoli R, Ebner H, Auricchio F (2010) Asleep-awake-asleep technique during carotid endarterectomy: a case series. J Cardiothorac Vasc Anesth 24:550-554. | 3 |
| Marcucci G, Siani A, Accrocca F, Gabrielli R, Giordano A, Antonelli R, Sbroscia A, Mounayergi F (2011) Preserved consciousness in general anesthesia during carotid endarterectomy: a six-year experience. Interact Cardiovasc Thorac Surg 13:601-605. | 3 |

**PICO 2**

| Evaluation of evidences. Comparisons among neurological monitoring techniques | |
| --- | --- |
| The following papers have been selected. In detail:  n. 6 Systematic review and meta-analysis  n. 2 Cohort studies | Level of evidence |
| **Systematic review and meta-analysis (n. 6)** | |
| Chongruksut W, Vaniyapong T, Rerkasem K (2014) Routine or selective carotid artery shunting for carotid endarterectomy (and different methods of monitoring in selective shunting). Cochrane Database Syst Rev. 23;2014(6):CD000190. | 2- |
| Guay J, Kopp S (2013) Cerebral monitors versus regional anesthesia to detect cerebral ischemia in patients undergoing carotid endarterectomy: a meta-analysis. Can J Anaesth 60:266-79. | 2+ |
| Chang R, Reddy RP, Sudadi S, Balzer J, Crammond DJ, Anetakis K, Thirumala PD (2020) Diagnostic accuracy of various EEG changes during carotid endarterectomy to detect 30-day perioperative stroke: A systematic review. Clin Neurophysiol 131:1508-1516. | 2+ |
| Nwachuku EL, Balzer JR, Yabes JG, Habeych ME, Crammond DJ, Thirumala PD (2015) Diagnostic value of somatosensory evoked potential changes during carotid endarterectomy: a systematic review and meta-analysis. JAMA Neurol 72:73-80. | 2+ |
| Udesh R, Natarajan P, Thiagarajan K, Wechsler LR, Crammond DJ, Balzer JR, Thirumala PD (2017) Transcranial doppler monitoring in carotid endarterectomy: A systematic review and meta-analysis. J Ultrasound Med 36:621-630. | 2+ |
| Thirumala PD, Thiagarajan K, Gedela S, Crammond DJ, Balzer JR (2016) Diagnostic accuracy of EEG changes during carotid endarterectomy in predicting perioperative strokes. J Clin Neurosci 25:1-9. | 2+ |
| **Cohort studies (n.2)** | |
| Malcharek MJ, Ulkatan S, Marinò V, Geyer M, Lladó-Carbó E, Perez-Fajardo G, Arranz-Arranz B, Climent J, Aloj F, Franco E, Chiacchiari L, Kulpok A, Sablotzki A, Hennig G, Deletis V (2013) Intraoperative monitoring of carotid endarterectomy by transcranial motor evoked potential: a multicenter study of 600 patients. ClinNeurophysiol 124:1025-1030. | 2- |
| Thirumala PD, Natarajan P, Thiagarajan K, Crammond DJ, Habeych ME, Chaer RA, Avgerinos ED, Friedlander R, Balzer JR (2016) Diagnostic accuracy of somatosensory evoked potential and electroencephalography during carotid endarterectomy. Neurol Res 38:698-705. | 2- |

**PICO 3**

| Evaluation of evidences. Comparisons between heparin neutralization strategy versus non-neutralization. | |
| --- | --- |
| The following papers have been selected. In detail:  n. 2 Systematic review and meta-analysis  n. 1 Randomized controlled trial | Level of evidence |
| **Systematic review and meta-analysis (n. 2)** | |
| Newhall KA, Saunders EC, Larson RJ, Stone DH, Goodney PP (2016) Use of protamine for anticoagulation during carotid endarterectomy: a meta-analysis. JAMA Surg 151:247-55. | 2+ |
| Kakisis JD, Antonopoulos CN, Moulakakis KG, Schneider F, Geroulakos G, Ricco JB (2016) Protamine reduces bleeding complications without increasing the risk of stroke after carotid endarterectomy: a meta-analysis. Eur J VascEndovascSurg 52:296-307. | 2+ |
| **Randomized controlled studies (n.1)** | |
| Fearn SJ, Parry AD, Picton AJ, Mortimer AJ, McCollum CN (1997) Should heparin be reversed after carotid endarterectomy? A randomised prospective trial. Eur J Vasc Endovasc Surg 13:394-7. | 1- |

**PICO 4**

| Evaluation of evidences. Comparisons between postoperative blood pressure monitoring versus non-monitoring | |
| --- | --- |
| The following papers have been selected. In detail:  n. 2 Systematic review and meta-analysis  n. 4 Case series  n. 3 Cohort studies  n.1 Narrative review | Level of evidence |
| **Systematic review and meta-analysis (n. 2)** | |
| Demirel S, Goossen K, Bruijnen H, Probst P, Böckler D (2017) Systematic review and meta-analysis of postcarotid endarterectomy hypertension after eversion versus conventional carotid endarterectomy. J Vasc Surg 65:868-882. | 2++ |
| Bouri S, Thapar A, Shalhoub J, Jayasooriya G, Fernando A, Franklin IJ, Davies AH (2011) Hypertension and the post-carotid endarterectomy cerebral hyperperfusion syndrome. Eur J VascEndovascSurg 41:229-37. | 2+ |
| **Case series (n.4)** | |
| Kotsis T, Christoforou P, Nastos K (2020) Carotid body baroreceptor preservation and control of arterial pressure in eversion carotid endarterectomy. Int J Angiol 29:33-383. | 3 |
| Ben Ahmed S, Daniel G, Benezit M, Bailly P, Aublet-Cuvelier B, Mulliez A, Ribal JP, Rosset E (2015) Does the technique of carotid endarterectomy determine postoperative hypertension? Ann Vasc Surg 29:1272-80. | 3 |
| Newman JE, Bown MJ, Sayers RD, Thompson JP, Robinson TG, Williams B, Panerai R, Lacy P, Naylor AR (2017) Post-carotid endarterectomy hypertension. Part 1: association with pre-operative clinical, imaging, and physiological parameters. Eur J Vasc Endovasc Surg 54:551-563. | 3 |
| Newman JE, Bown MJ, Sayers RD, Thompson JP, Robinson TG, Williams B, Panerai R, Lacy P, Naylor AR (2017) Post-carotid endarterectomy hypertension. Part 2: association with peri-operative clinical, anaesthetic, and transcranial doppler derived parameters. Eur J Vasc Endovasc Surg 54:564-572. | 3 |
| **Cohort studies (n.3)** | |
| Lee S, Conway AM, Nguyen Tranh N, Anand G, Leung TM, Fatakhova O, Giangola G, Carroccio A (2020) Risk Factors for Postoperative Hypotension and Hypertension following Carotid Endarterectomy. Ann Vasc Surg 69:182-189. | 2- |
| Naylor AR (2017) Medical treatment strategies to reduce perioperative morbidity and mortality after carotid surgery. SeminVasc Surg 30:17-24. | 2+ |
| Naylor AR, Sayers RD, McCarthy MJ, Bown MJ, Nasim A, Dennis MJ, London NJ, Bell PR (2013) Closing the loop: a 21-year audit of strategies for preventing stroke and death following carotid endarterectomy. Eur J Vasc Endovasc Surg 46:161-70. | 2- |
| **Narrative review (n.1)** | |
| Wang GJ, Beck AW, DeMartino RR, Goodney PP, Rockman CB, Fairman RM (2017) Insight into the cerebral hyperperfusion syndrome following carotid endarterectomy from the national Vascular Quality Initiative. J Vasc Surg 65. 381-389.e2. | 4 |
